# Supplementary material for: Smooth Interpolating Curves with Local Control and Monotone Alternating Curvature
Source: Comput Graph Forum. 2022 Oct 6;41(5):25–38. doi: 10.1111/cgf.14600 (PMC9827861; doi:10.1111/cgf.14600)
Supplement: Supplementary file 1 — Supplement Material [file CGF-41-25-s001.zip › Local-Smooth-Interpolating-MonoCurvature/extern/clothoids/submodules/Utils/docs/api-cpp/root.html]

C++ API — Utils v1.2

### Navigation

- index
- toc
- next
- previous
- Utils »
- C++ API

# C++ API¶

## Class Hierarchy¶

- - Namespace detail
    - Template Struct basic\_data
  - Namespace rang
    - Enum bg
    - Enum bgB
    - Enum control
    - Enum fg
    - Enum fgB
    - Enum style
    - Enum winTerm

## File Hierarchy¶

- - Directory Utils
    - File Console.hxx
    - File Malloc.hxx
    - File Numbers.hxx
    - File Quaternion.hxx
    - File rang.hxx
    - File Table.hxx
    - File ThreadPool.hxx
    - File TicToc.hxx
    - File Token.hxx
    - File Trace.hxx
    - File Utils.hxx
  - File Console.cc
  - File CPUinfo.cc
  - File format.cc
  - File Malloc.cc
  - File Numbers.cc
  - File os.cc
  - File rang.cc
  - File Table.cc
  - File TicToc.cc
  - File Token.cc
  - File Trace.cc
  - File Utils.cc
  - File Utils.hh
  - File Utils\_CPU\_info.hh
  - File Utils\_zstr.hh

## Full API¶

### Namespaces¶

- Namespace @5

- Namespace detail
  - Classes
  - Functions

- Namespace rang
  - Enums

- Namespace Utils
  - Detailed Description
  - Functions

### Classes and Structs¶

- Template Struct basic\_data
  - Struct Documentation

### Enums¶

- Enum bg
  - Enum Documentation

- Enum bgB
  - Enum Documentation

- Enum control
  - Enum Documentation

- Enum fg
  - Enum Documentation

- Enum fgB
  - Enum Documentation

- Enum style
  - Enum Documentation

- Enum winTerm
  - Enum Documentation

### Functions¶

- Template Function detail::format\_float
  - Function Documentation

- Function detail::locale\_ref::get< std::locale >
  - Function Documentation

- Function Utils::cpuInfo
  - Function Documentation

- Function Utils::has\_3Dnow
  - Function Documentation

- Function Utils::has\_3DnowExt
  - Function Documentation

- Function Utils::has\_MMX
  - Function Documentation

- Function Utils::has\_MMXplus
  - Function Documentation

- Function Utils::has\_SSE
  - Function Documentation

- Function Utils::has\_SSE2
  - Function Documentation

- Function Utils::has\_SSE3
  - Function Documentation

- Function Utils::has\_SSE41
  - Function Documentation

- Function Utils::has\_SSE42
  - Function Documentation

- Function Utils::has\_SSE4a
  - Function Documentation

- Function Utils::has\_SSE5
  - Function Documentation

- Function Utils::has\_SSSE3
  - Function Documentation

- Function Utils::info
  - Function Documentation

### Variables¶

- Variable instantiate\_format\_float
  - Variable Documentation

### Defines¶

- Define FMT\_ARGS
  - Define Documentation

### Quick search

### Table of Contents

- rang - Colors for your Terminal
- Zstr
- C++ API
  - Class Hierarchy
  - File Hierarchy
  - Full API

«
hide menu

menu
sidebar
»

### Navigation

- index
- toc
- next
- previous
- Utils »
- C++ API

© Copyright 2021, Enrico Bertolazzi.
Created using Sphinx 4.2.0.
